# Supplementary material for: Serum Erythroferrone Levels Associate with Mortality and Cardiovascular Events in Hemodialysis and in CKD Patients: A Two Cohorts Study
Source: J Clin Med. 2019 Apr 16;8(4):523. doi: 10.3390/jcm8040523 (PMC6518296; doi:10.3390/jcm8040523)
Supplement: Supplementary file 1 [file jcm-08-00523-s001.pdf]

Table S1. Causes of death in the two study cohorts

Causes of death	HD cohort n (%)	CKD cohort n (%)
Stroke	54 (11.3)	4 (9.5)
Myocardial infarction	49 (10.3)	6 (14.3)
Heart failure	59 (12.4)	2 (4.8)
Sudden death	69 (14.5)	13 (30.9)
Pulmonary embolism	3 (0.6)	1 (2.4)
Peripheral vascular disease	4 (0.8)	-
Sepsis/Severe infection	48 (10.2)	2 (4.8)
Neoplasia and Cachexia	94 (19.7)	7 (16.6)
Respiratory failure	4 (0.8)	-
Liver and gastrointestinal diseases	23 (4.8)	-
Severe hemorrhage	18 (3.8)	-
Other causes	40 (8.3)	5 (11.9)
Unknown	12 (2.5)	2 (4.8)
Total	477 (100)	42 (100)

Table S2. Adjusted association between serum ERFE and mortality and non-fatal CV events in the HD patients with ERFE levels >1.59 ng/mL

	**Unit of increase**	**Hazard ratio (95% CI), P value**
ERFE	5 ng/mL	**1.04 (1.01-1.07), P=0.013**
Age	1 year	1.03 (1.03-1.04), P<0.001
Male sex		1.13 (0.93-1.38), P=0.22
Smoke	yes/no	1.09 (0.83-1.44), P=0.54
Diabetes	yes/no	1.35 (1.09-1.68), P=0.006
Cholesterol	1 mg/dL	0.99 (0.99-1.00), P=0.03
Systolic blood pressure	1 mmHg	1.01 (1.00-1.01), P=0.04
Background CV comorbidities	yes/no	1.46 (1.20-1.77), P<0.001
Hemoglobin	1 g/dL	0.99 (0.93-1.05), P=0.71
C-reactive protein	25 mg/L	1.02 (0.97-1.07), P=0.50
Ferritin	50 ng/mL	1.02 (1.01-1.03), P=0.001
Iron	1 μg%	0.99 (0.99-1.01), P=0.27
BMI	1 kg/m^2	0.99 (0.97-1.02), P=0.77
Albumin	1 g/dL	0.65 (0.53-0.81), P<0.001
Phosphate	1 mg/dL	1.08 (1.02-1.15), P=0.013
Parathyroid hormone	50 pg/mL	1.01 (0.99, 1.02), P=0.23
Dialysis vintage	1 month	1.01 (1.00-1.01), P=0.001
Kt/V	1 unit	0.94 (0.73-1.22), P=0.65

Table S3. Adjusted association between serum ERFE and mortality and non-fatal CV events in the CKD patients not on ESA treatment

	Unit of increase	Hazard ratio (95% CI), P value
ERFE	2 ng/mL	**1.06 (1.03-1.09), P<0.001**
Age	1 year	1.08 (1.05-1.11), P<0.001
Smoke	yes/no	1.85 (1.22-2.79), P=0.004
Background CV comorbidities	yes/no	2.35 (1.59-3.49), P<0.001
Albumin	1 g/dL	0.65 (0.41-1.03), P=0.07
Phosphate	1 mg/dL	1.23 (0.97-1.58), P=0.09
Hemoglobin	1 g/dL	0.83 (0.73-0.95), P=0.006
Parathyroid hormone	50 pg/mL	0.87 (0.75-1.02), P=0.09

Variables out of the model: male sex (P=0.20), diabetes (P=0.19), GFR (P=0.99), cholesterol (P=0.94), urinary protein (P=0.18), ferritin (P=0.67), iron (P=0.80), BMI (P=0.55), systolic blood pressure (P=0.11), C-reactive protein (P=0.21).

Table S4. Adjusted association between serum ERFE and mortality and non-fatal CV events in the CKD patients not on ESA treatment and with ERFE levels >1.59 ng/mL

	**Unit of increase**	**Hazard ratio (95% CI), P value**
ERFE	2 ng/mL	**1.06 (1.03-1.10), P<0.001**
Age	1 year	1.08 (1.03-1.12), P=0.001
Smoke	yes/no	2.04 (1.19-3.50), P=0.009
Background CV comorbidities	yes/no	2.79 (1.65-4.72), P<0.001
Albumin	1 g/dL	0.46 (0.25-0.82), P=0.009
Hemoglobin	1 g/dL	0.68 (0.58-0.81), P<0.001
Parathyroid hormone	50 pg/mL	0.83 (0.67-1.03), P=0.09

Variables out of the model: male sex (P=0.73), diabetes (P=0.58), GFR (P=0.53), cholesterol (P=0.45), urinary protein (P=0.44), ferritin (P=0.76), iron (P=0.78), BMI (P=0.99), systolic blood pressure (P=0.12), C-reactive protein (P=0.11), phosphate (P=0.14).

Figure S1: Serum ERFE levels in the HD and in the CKD cohort. Data in males and females are separately presented.

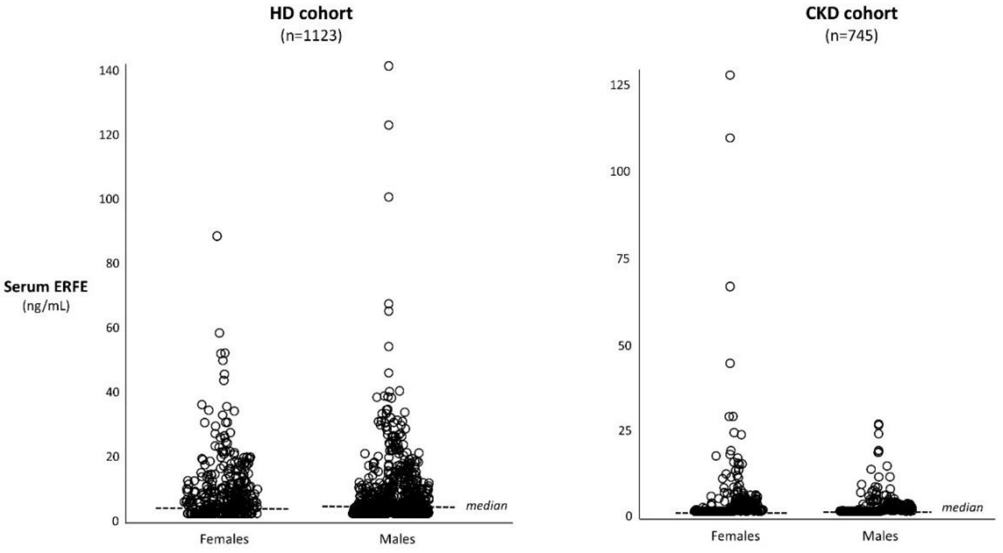

Supplementary Figure 1
